# Effect of the SOS Response on the Mean Fitness of Unicellular Populations: A Quasispecies Approach

**DOI:** 10.1371/journal.pone.0014113

**Published:** 2010-11-30

**Authors:** Amit Kama, Emmanuel Tannenbaum

**Affiliations:** Department of Chemistry, Ben-Gurion University of the Negev, Be'er-Sheva, Israel; University of California, United States of America

## Abstract

The goal of this paper is to develop a mathematical model that analyzes the selective advantage of the SOS response in unicellular organisms. To this end, this paper develops a quasispecies model that incorporates the SOS response. We consider a unicellular, asexually replicating population of organisms, whose genomes consist of a single, double-stranded DNA molecule, i.e. one chromosome. We assume that repair of post-replication mismatched base-pairs occurs with probability 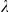, and that the SOS response is triggered when the total number of mismatched base-pairs is at least 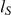. We further assume that the per-mismatch SOS elimination rate is characterized by a first-order rate constant 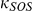. For a single fitness peak landscape where the master genome can sustain up to 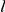 mismatches and remain viable, this model is analytically solvable in the limit of infinite sequence length. The results, which are confirmed by stochastic simulations, indicate that the SOS response does indeed confer a fitness advantage to a population, provided that it is only activated when DNA damage is so extensive that a cell will die if it does not attempt to repair its DNA.

## Introduction

Genetic repair is an essential component of cellular genomes. Without mechanisms for repairing damaged and mutated DNA, genomes could not achieve sufficient information content to code for the variety and complexity of modern terrestrial life [1].

Genetic repair mechanisms fall into two main categories: Those that correct base mis-pairings during the replication cycle of a cell, and those that repair mutated and damaged DNA during the growth (G) phase of the cellular life cycle [1].

Two important examples of the first class of repair mechanisms are DNA proofreading and mismatch repair (MMR). DNA proofreading is a repair mechanism that is built into the DNA replicases themselves. During daughter strand synthesis, an erroneously matched base is excised, and a second attempt at a base pairing is made [1]. Mismatch repair also removes erroneous bases from the daughter strand, but does this shortly after daughter strand synthesis [1].

Two important examples of the second class of repair mechanisms are Nucleotide Excision Repair (NER) and the SOS response [1]. NER protects a cell from damage due to radiation, chemical mutagens, and metabolic free radicals by removing damaged portions of the DNA strand and using the other, presumably undamaged strand as a template for re-synthesis of the excised region [1].

The SOS response is a genomic repair mechanism that only activates when there is extensive damage to the cellular genome. When DNA damage is sufficiently extensive, the cell stops growing, and the SOS repair pathways attempt to restore complementarity to the genome [1]. The SOS response only takes effect when DNA damage is so extensive that it may be impossible to use undamaged template strands to correctly re-synthesize damaged portions of the genome. Thus, although this means that the SOS repair mechanism is highly error prone, it is evolutionary advantageous for the cell to repair the genome and risk fixing deleterious mutations, than it is to leave the damaged genome unrepaired [1]–[4].

In recent work with quasispecies models of evolutionary dynamics, quasispecies models [5]–[7] considering the first class of repair mechanisms have been studied [8]–[11]. In addition, semiconservative replication, including semiconservative replication with imperfect lesion repair (i.e. not all base-pair mismatches are eliminated), has been considered [12]–[15]. Additional effects, such as multiply-gened genomes, as well as multiply chromosomed genomes, have been considered as well [16], [17].

This paper continues the theme of incorporating various details characteristic of cellular genomes by developing a quasispecies model that takes into consideration the SOS repair mechanism. The model is highly simplified, and therefore only a first step in developing proper evolutionary dynamics equations with SOS repair. Nevertheless, because our model is analytically tractable, we believe it is a useful and important initial approach to mathematically modeling the evolutionary aspects of the SOS repair pathway. A proper modeling of the SOS response is an essential component of developing a quantitative theory of mutation-propagation in cellular organisms, which is important for understanding phenomena such as the emergence of antibiotic drug resistance in bacteria, and cancer in multicellular organisms [2]–[4].

## Materials and Methods

### Definitions and Model Set-Up

We consider a unicellular population of asexually replicating organisms, whose genomes consist of a single DNA molecule, i.e. one chromosome. The genome may then be denoted by 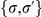, where 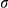, 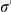 denote the two strands of the DNA molecule. If the genome is of length 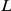, then we may write 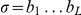, 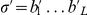 where each base 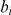, 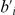 is chosen from an alphabet of size 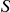 (usually 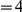). If 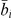 denotes the base complementary to 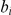 (for the standard Watson-Crick bases, the pairings are 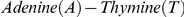, 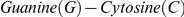), and 
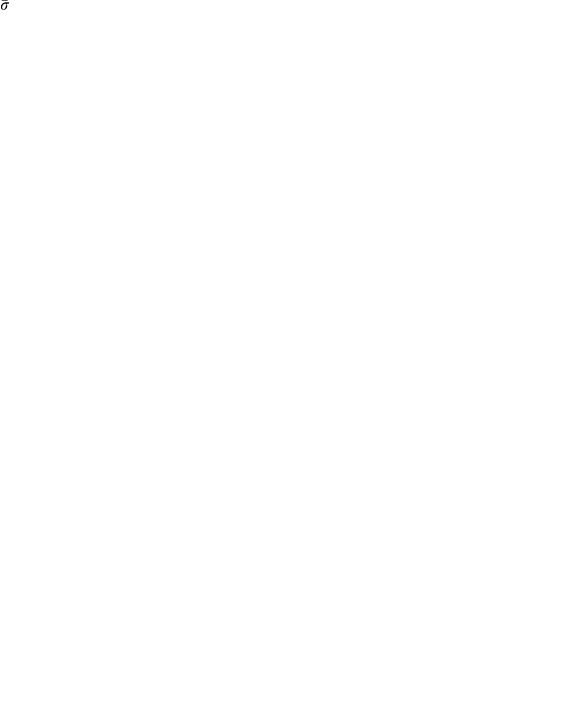
 denotes the strand complementary to 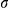, then 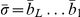. This follows from the antiparallel nature of double-stranded DNA [1].

We let 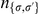 denote the number of organisms with genome 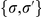, and we assume that replication occurs with a genome-dependent, first-order rate constant, denoted 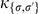. The set of all 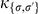 defines the *fitness landscape*. It should be emphasized that fitness in this model only refers to replication rate. In particular, this model does not consider cell death.

The semiconservative replication of the DNA genomes happens in three stages:

1. Strand separation, whereby each strand of the chromosome separates to act as a template for daughter strand synthesis.
2. Daughter strand synthesis. We assume a genome and base-independent mismatch probability 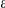. This error probability 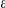 includes all error correction mechanisms, such as proofreading and mismatch repair, that are active during the replication phase of the cell.
3. Lesion repair, where any post-replication mismatches are removed. Here, there is no longer the parent-daughter strand discrimination that was available during daughter strand synthesis, so in contrast to DNA proofreading and mismatch repair, lesion repair has a 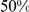 chance of removing the mutation, and a 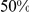 chance of communicating it to the parent strand and fixing the mutation in the genome (the lesion repair can occur via either Base or Nucleotide Excision Repair) [1]. We also do not assume that lesion repair is perfectly efficient, so that we consider a genome and base-independent probability 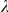 of removing a mismatch. We call 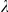 the lesion repair efficiency.

**Figure 1:**
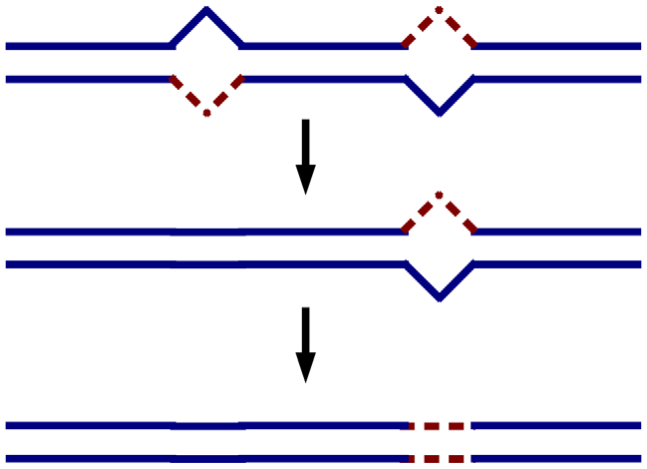
(Color online) Illustration of the SOS repair mechanism being considered in this paper. A DNA genome with two base-pair mismatches is restored to a fully complementary genome in two repair steps, where during each step a single mismatch (i.e. lesion) is eliminated. The first lesion is repaired correctly, so that the original base-pair of the master genome strands (solid blue lines) is restored, while the second lesion is repaired incorrectly, so that a mutation (dotted red lines) becomes fixed in the genome.

In our simplified model, the SOS response is triggered if a given genome has at least 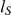 mismatches. The replication rate of all cells undergoing SOS repair is zero. We assume that removal of mismatches is catalyzed by an enzyme that binds to a mismatch and then eliminates the mismatch at a rate characterized by a first-order rate constant 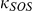. Therefore, the probability that a given mismatch is eliminated over an infinitesimal time interval 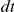 is given by 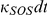 (see Figure 1).

In this paper, we will consider the behavior of the model in the limit of infinite sequence length. If 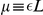 is held constant as 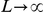, then the probability of an error-free daughter strand synthesis is given by 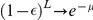. Therefore, fixing 
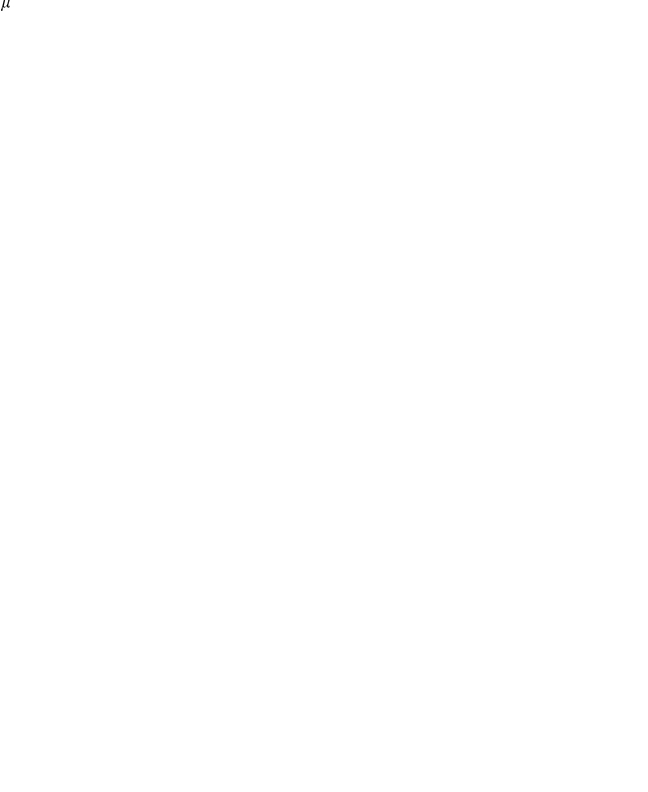
 in the infinite sequence length limit is equivalent to fixing the per-genome replication fidelity. It should be noted that 
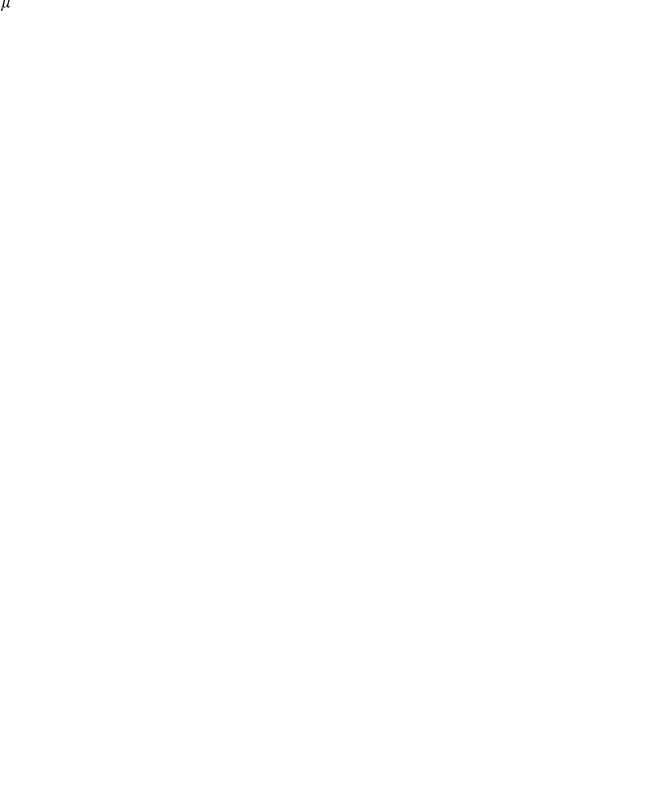
 is the average number of mismatches produced per DNA strand per replication cycle.

The assumption of infinite sequence length is a common assumption in quasispecies theory, because it is the mathematical formalization of the long genome-length regime that makes the neglect of backmutations exact. While finite genome length effects need to be considered in dynamic fitness landscapes, where adaptation to specific genomes is important [18], for static landscapes (like the one being considered in this paper), good agreement with the infinite sequence length results may be obtained with genomes as short as ten bases.

Finally, we assume that the fitness landscape is defined by a master genome 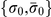. Specifically, we define a genome 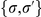 to be viable, with a first-order growth rate constant 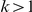, if it has fewer than 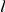 mismatches, and if it does not differ from 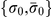 by any fixed mutations. Otherwise, the genome is unviable, with a first-order growth rate constant of 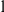. We recognize that this terminology is somewhat inappropriate, since a genome with a first-order growth rate constant of 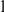 may still replicate. However, this is standard terminology from quasispecies theory, where “viable” and “unviable” are taken to be synonymous with “higher fitness” and “lower fitness” respectively.

The justification for this choice of fitness landscape is as follows: If a genome has a fixed mutation, then neither DNA strand corresponds to either of the master strands 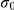, 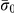. As a result, the genome does not contain all of the information corresponding to a viable organism, hence the organism is unviable. While this assumption is clearly extreme and oversimplified, it is the analogue of the single-fitness-peak landscape for single-stranded genomes.

However, in the case of a mismatch where one of the bases is the same as the corresponding base in one of the master strands, the information contained in the master genome is still preserved in one of the strands, so that the organism is assumed to remain viable if the total number of such mismatches does not exceed some cutoff value 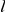.

For convenience, Table 1 summarizes the main parameters of the model.

**Table 1:** The various parameters and their definitions in our model.

Parameter	Definition
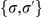	General notation for a genome
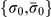	The master genome
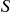	Alphabet size
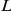	Genome length
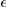	Per-base mismatch probability during daughter strand synthesis
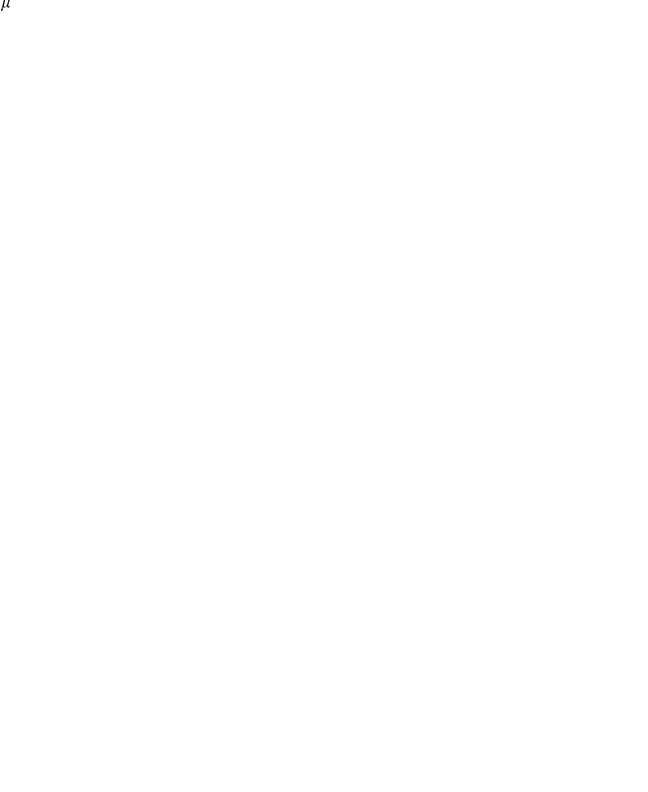	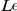
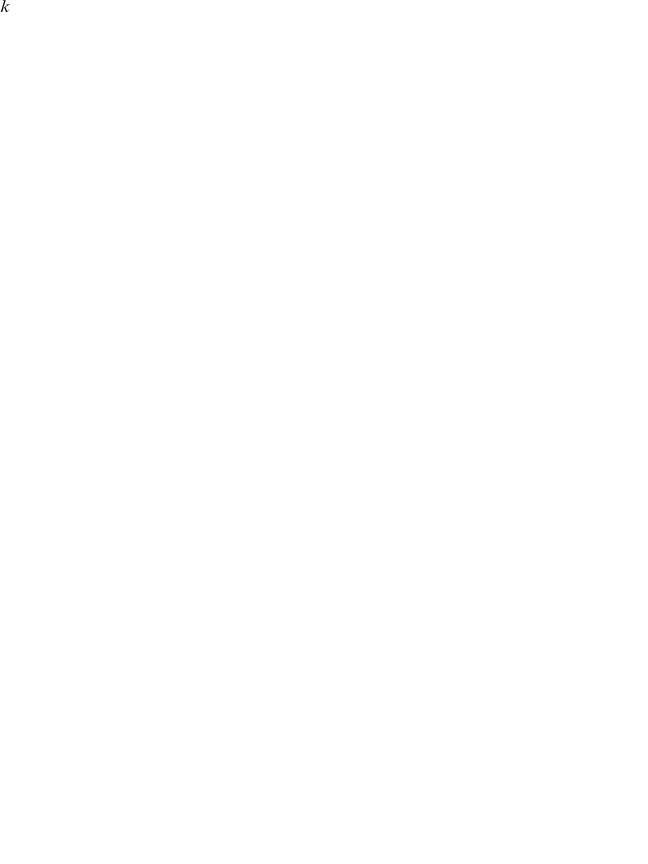	Fitness of the master genome
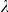	Lesion repair probability
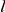	Maximum number of mismatches a genome can tolerate and still remain viable
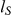	The minimum number of mismatches required to trigger the SOS response
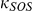	First-order rate constant characterizing the rate of SOS repair

### Symmetrized Population Distribution

We can develop the infinite sequence length equations for our model, assuming an initially prepared clonal population consisting entirely of the wild-type (mutation-free) genome 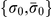, i.e. a population consisting entirely of the fastest replicating genotype. Because, during replication, only a finite number of mutations are possible, at any time time the population will consist of a distribution of genomes 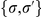 where 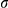, 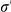 differ from either 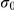 and 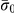 in at most a finite number of spots. Thus, given two gene sequences 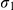, 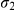, if we let 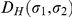 denote the Hamming distance [19] between 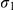 and 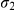 (i.e. the number of sites where 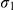 and 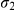 differ), then either 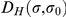 and 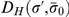 are finite, or 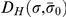 and 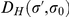 are finite.

The Hamming distance between two sequences 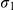 and 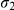 is simply equal to the number of positions by which they differ. It may be readily shown that the Hamming distance is a metric over the space of sequences [19], so that, in particular, the Hamming distance satisfies the Cauchy-Schwartz Inequality: Given three sequences 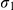, 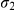, and 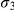, we have 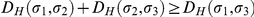.

Now, in the limit of infinite sequence length, it may be shown that, with probability 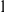, that the Hamming distance between 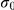 and its complement 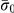 is infinite [12]. Therefore, if 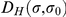 and 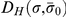 were both finite, we would obtain 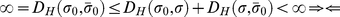, and so 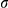 cannot simultaneously be of finite Hamming distance to 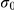 and 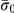. Similarly, 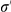 cannot simultaneously be of finite Hamming distance to 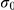 and 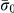.

As a result, we can define a strand ordering 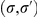 for a genome 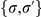, where it is understood that 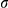 is a finite Hamming distance from 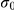 and 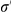 is a finite Hamming distance from 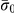.

A given genome 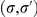 may then be characterized by four parameters 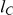, 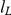, 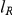, and 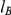. We let 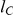 denote the number of sites where 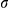 and 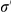 are both complementary, yet differ from the corresponding bases in 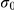 and 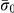. We let 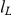 denote the number of sites where 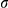 differs from 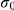, but 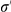 is identical to 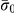. We let 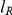 denote the number of sites where 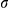 is identical to 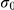, but 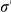 differs from 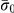. Finally, we let 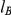 denote the number of sites where 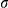 and 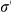 differ from 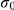 and 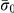, but are not complementary (for an illustration of these parameters, see [7], [13]).

Note that the fitness landscape depends only on 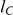, 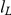, 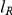, and 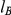, and hence the fitness of a given organism may be denoted by 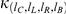, where for our single-fitness-peak landscape we have 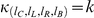 if 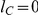 and 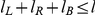, and 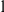 otherwise. The condition 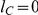 means that there are no mutations fixed in the genome, while the condition 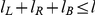 means that there are fewer than 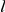 lesions.

By the symmetry of the fitness landscape, and by the symmetry of the initial population distribution, we can group all genomes of identical 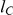, 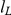, 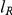, and 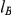, and derive the dynamical equations of the symmetrized population distribution. We therefore let 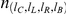 denote the total number of organisms in the population whose genomes are characterized by the parameters 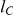, 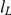, 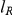, and 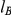, and we let 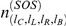 denote the total number of organisms in the population undergoing the SOS response, whose genomes are similarly characterized by the parameters 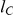, 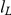, 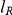, and 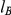. The corresponding population fractions are denoted 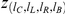 and 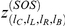, respectively.

### Dynamical Equations

To develop the dynamical equations for both the 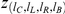 and the 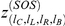 quantities, we begin by considering a genome 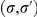, characterized by the parameters 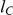, 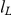, 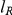, and 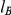.

We first consider the case where this genome is not undergoing the SOS response. Then, due to the semiconservative nature of DNA replication, this genome is being destroyed at a rate given by 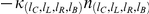. This genome, however, is produced by other genomes in the population, as a result of replication. So, consider some other genome 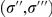 which produces 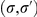 upon replication. This can either occur via the 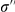 template strand, the 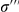 template strand, or both.

If the 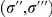 genome is characterized by the parameters 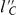, 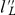, 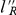, and 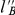, then 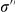 differs from 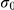 in 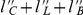 bases. Because sequence lengths are infinite, the probability of a mismatch in one of these bases during daughter strand synthesis is 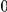. In the remaining sites, let 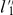 denote the number of mismatches that are not corrected, and 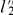 denote the number of mismatches that are repaired, but fixed as a mutation in the genome. Then the resulting genome 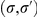 is characterized by:

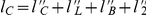

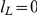

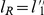

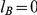

The probability of a given set of mutations corresponding to 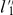, 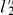, is 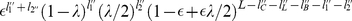. The term 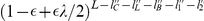 arises as a probability that the remaining 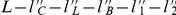 sites on 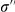 remain identical to 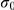, and the corresponding daughter strand sites are identical to 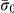. The per-site probability of this is the probability of error-free daughter strand synthesis, 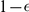, plus the probability of a mismatch, times 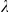, the probability that complementarity is restored, times 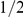, the probability that complementarity is restored correctly. It is assumed that complementarity is restored by various DNA repair mechanisms, such as Nucleotide Excision Repair (NER) and Base Excision Repair (BER) [1]. However, because NER and BER do not distinguish between parent and daughter strands, the probability of correctly removing a mismatch via these mechanisms is 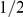.

The degeneracy is given by 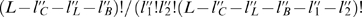, so in the limit of infinite sequence length the total probability becomes,
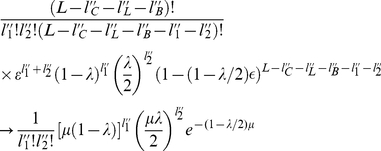
(1)


If 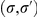 is generated by 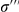, then we have,

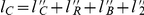

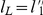

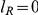

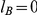

We also obtain an overall transition probability of 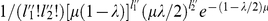.

It is important to note from the 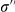 and 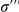 results that genomes with 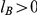 cannot be generated during replication. Since SOS repair eliminates mismatches, it follows that a population where 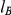 is initially 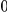 for all genomes will always have a population where 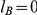. Therefore, we may assume in subsequent derivations that 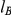, 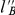 are 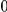.

Furthermore, note that strands 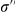 that are a finite Hamming distance away from 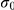 can only generate daughter genomes where 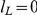, while strands 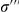 that are a finite Hamming distance away from 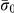 can only generate daughter genomes where 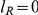.

Then for the genomes 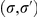 generated by 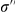, we have 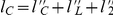, and 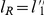. Therefore, the restriction on 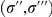 is that 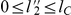, 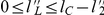, and 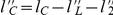. Note that there is no restriction on 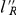.

Then for the population number 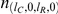, we have a contribution from the 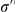 strands of
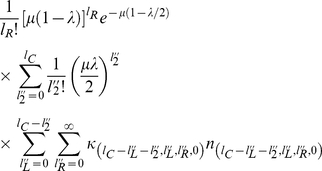
(2)


A similar expression is obtained for the population number 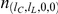, except 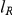 is replaced with 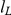, and the roles of 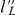 and 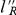 are exchanged.

It should also be noted that, by the symmetry of the fitness landscape, we have that 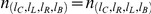. Another way to note this is that, for a given genome 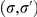, if we change the ordering of the strands so that the first strand is of finite Hamming distance to 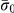, and the second strand is of finite Hamming distance to 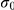, then the genome 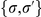 must be represented as 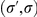, and is characterized by the parameters 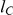, 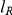, 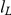, and 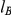. If 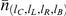 denotes the number of genomes characterized by 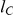, 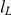, 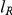, and 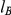, with respect to the 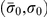 strand ordering, then since there is a one-to-one correspondence between genomes 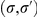 with parameters 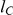, 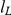, 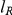, 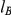 with respect to the first ordering, and genomes 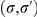 with parameters 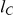, 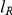, 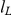, 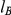 with respect to the second ordering, it follows that 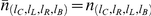. However, since the fitness landscape is invariant under strand ordering, we have 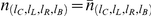, so that 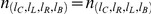.

Taking into consideration the contribution to 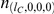, we may put everything together and obtain, after changing variables from population numbers to population fractions, the differential equations governing the time evolution of the various population fractions. These equations are,
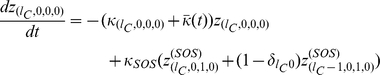


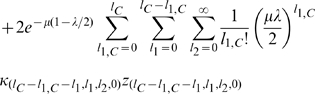


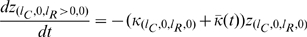


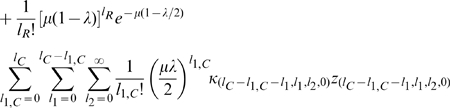


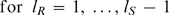


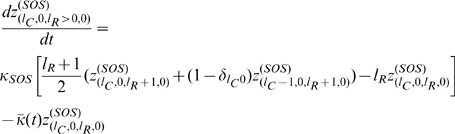


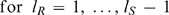


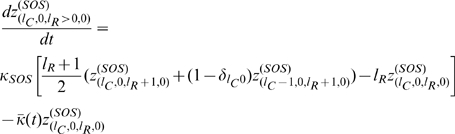


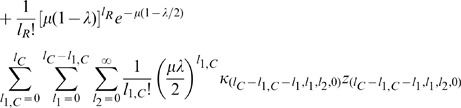


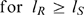
(3)where 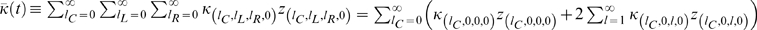 is the mean fitness of the population. It should also be noted that 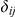 is the Kronecker delta function, so that 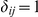 if 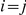, and 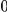 otherwise.

Note that we do not write down the dynamical equations for 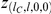 or 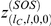, since they are redundant.

The factor of 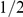 appearing in the SOS terms arises from the fact that when a mismatch is removed, it either corrects the daughter strand synthesis error, or it fixes the mismatch as a mutation in the genome. In the former case, the value of 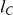 remains unchanged, while in the latter case it is incremented by 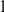.

It should be noted that this factor is missing in the contribution to 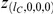 from SOS repair. The reason for this is that this contribution comes from 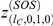, 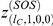, 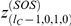, and 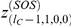. However, because 
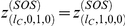
, and 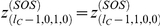, we may combine identical terms and eliminate the factor of 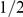.

The factor of 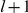 and 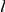 in front of the 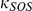 rate constant arises from the fact that the fraction of genomes whose SOS enzymes are bound to a mismatch is proportional to the total number of mismatches, hence the resulting SOS rate constant is proportional to the total number of mismatches.

## Results and Discussion

### Steady-State Behavior

#### Definitions and basic equations

To obtain the steady-state behavior of our model, we begin by introducing some definitions that will allow us to simplify the calculations.

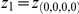
.

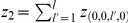
.

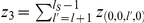
.

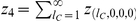
.

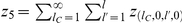
.

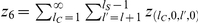
.

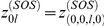
.

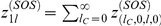
.

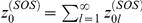
.

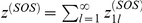
.

where we set 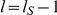 whenever 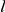 was previously defined as 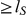. The differential equations for 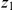, 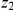, 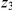, 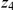, 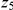, and 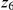 are readily derived. From the equations,
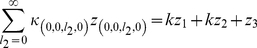
(4)and
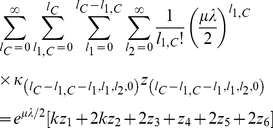
(5)we obtain,
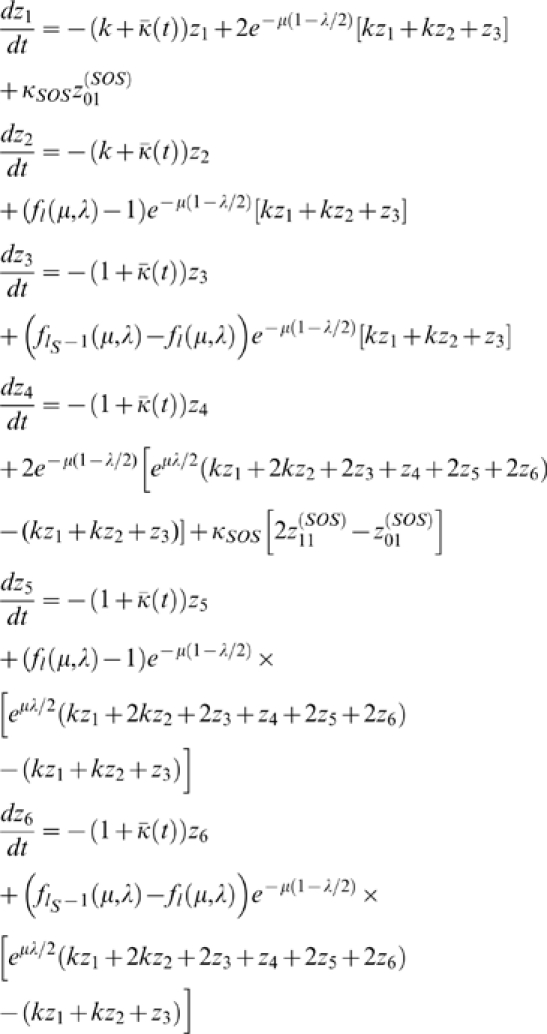
(6)We also have,
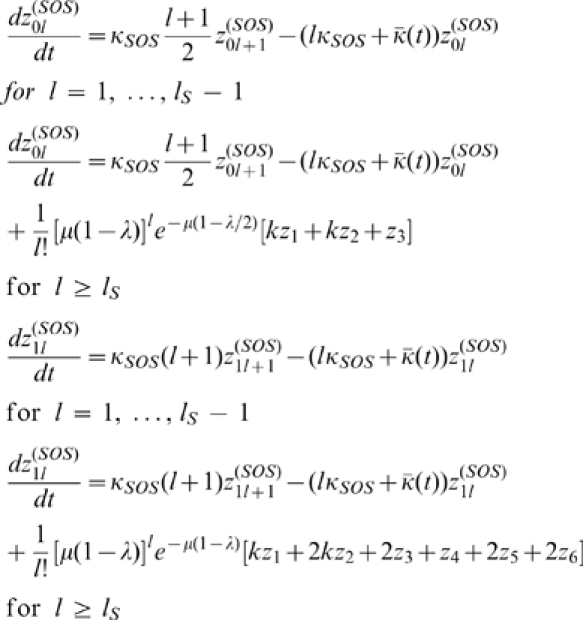
(7)


We can add these equations to obtain,
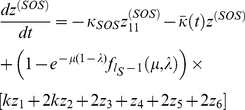
(8)


For the purposes of computing the mean fitness at steady-state, we can simplify the system of equations somewhat by defining 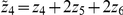. We obtain,
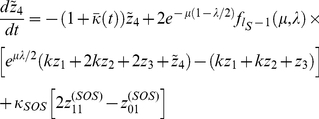
(9)For consistency of notation, in what follows we shall simply let 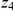 denote 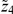.

#### Determining the population fractions 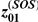, 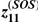, and 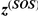


To obtain the steady-state behavior of this system of equations, we begin by first solving for the steady-state of the population undergoing SOS repair.

For 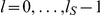 we have at steady-state that,
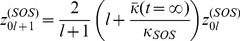
(10)which gives,
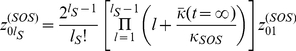
(11)


For 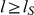, we have,
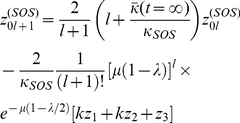
(12)


This expression has the form of the recursion relation, 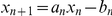. Using mathematical induction, it is possible to prove that 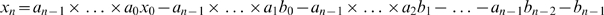. Therefore,
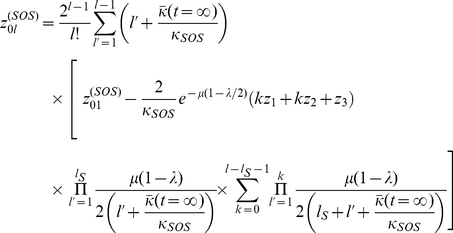
(13)where we define 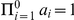.

If we define 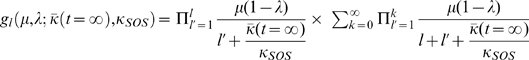, then imposing the requirement that 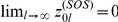 gives, at steady-state, that,
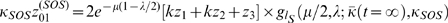
(14)


Using a similar argument, we obtain,
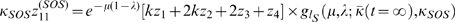
(15)


For the steady-state value of 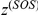, we have, using the identity 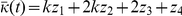,
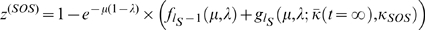
(16)


#### Computing the steady-state mean fitness 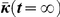


Plugging our expressions for 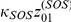 and 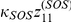 into the steady-state population fractions equations, we obtain,
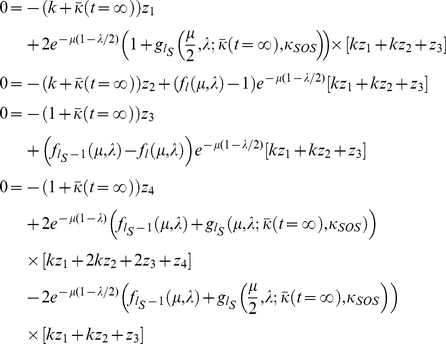
(17)


From these equations we may derive the equality,
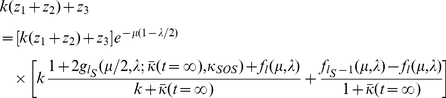
(18)Below the error catastrophe, when 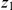, 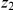, 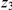 are not all 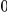, we may cancel 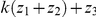 from both sides of the equation and re-arrange to obtain,
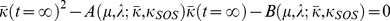
(19)where,
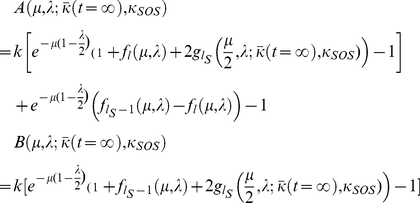
(20)


Beyond the error catastrophe, the mutation rate is sufficiently high that the selective advantage for remaining localized about the 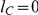 genomes disappears, so that 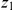, 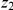, and 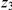 drop to 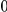. The relevant steady-state equation is then,
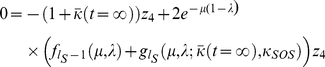
(21)which may be solved for 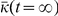 to give,
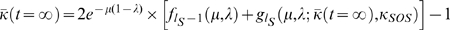
(22)


The error catastrophe occurs at the mutation rate for which the two expressions for the mean equilibrium fitness become equal. As with previous quasispecies models, the error catastrophe here also corresponds to a localization to delocalization transition over sequence space [5]–[7].

#### Limiting Cases

We now proceed to consider the behavior of the steady-state mean fitness for a number of limiting cases, in order to better understand our model. We consider the following cases: (1) 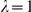, corresponding to perfect lesion repair, so that there are non-complementary genomes in the population. (2) 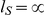, corresponding to the case where no genome ever undergoes the SOS response. (3) 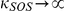, corresponding to the case where SOS repair happens rapidly, so that there is a negligible fitness penalty associated with undergoing the SOS response.

Case 1: 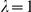 When 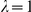, we get for 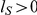 that 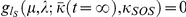, and that 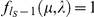. Therefore, above the error catastrophe, we obtain 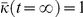. Below the error catastrophe, we have 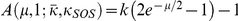, 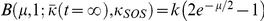, giving 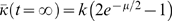. These results are in agreement with the solution of the semiconservative quasispecies equations with perfect lesion repair [12].

Case 2: 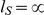 When 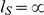, then 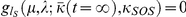. Below the error catastrophe, we have 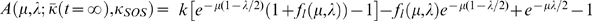, and 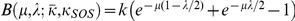. Above the error catastrophe, we have 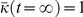. Both results are in agreement with the semiconservative quasispecies equations with arbitrary lesion repair efficiency [13].

Case 3: 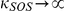 When 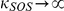, then 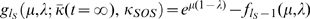. Above the error catastrophe, we get that 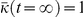. Below the error catastrophe, we obtain that, 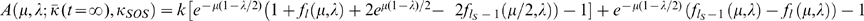, and 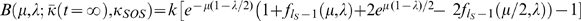.

Taking 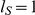 for 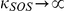 gives 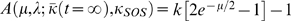, and 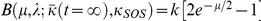, so that 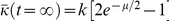 below the error catastrophe. This result is identical with the semiconservative quasispecies equations with perfect lesion repair, which makes sense, since here we assume that any lesion is eliminated instantaneously [13].

#### Optimal cutoff

If we assume that 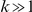, and 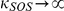, then it is possible to find the value of 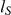 which maximizes the steady-state mean fitness 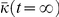. To do this, we define a normalized mean fitness 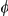 to be equal to 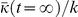, and if we divide Eq. (19) by 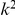, we obtain that 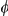 is the solution to,
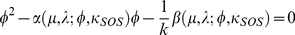
(23)where, 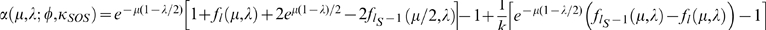, and 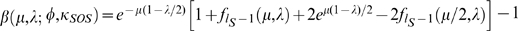.

Therefore, for large 
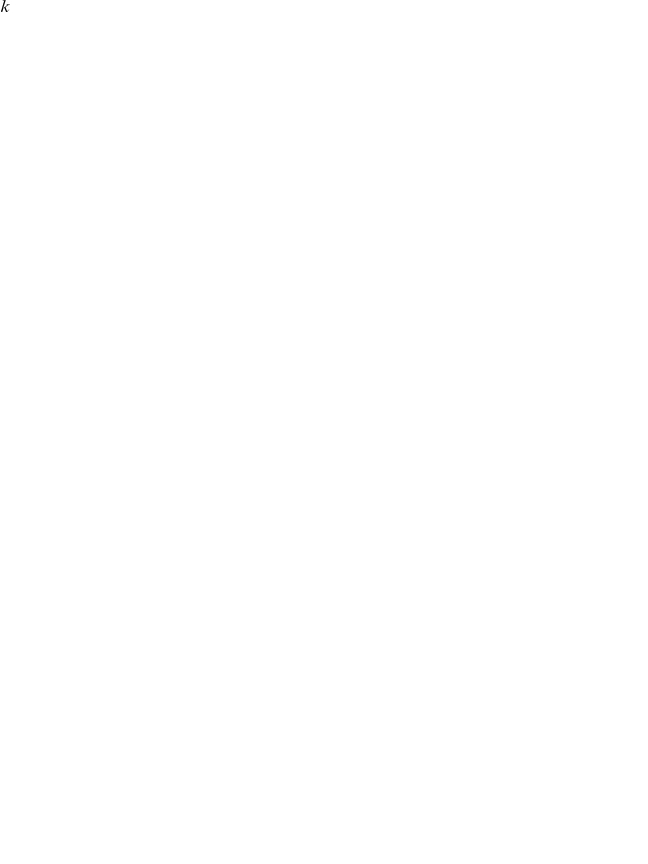
 we obtain that 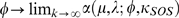, which gives,
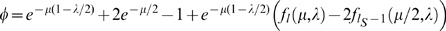
(24)so that maximizing 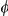 is equivalent to maximizing 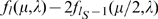.

Now, because 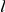 must be re-set to 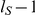 whenever we take 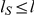, we can only vary 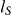 independently of 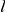 whenever 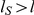. In this regime, the expression 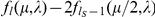 is maximized whenever 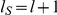.

In the regime where 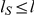, 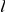 is re-set to 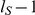, and so,
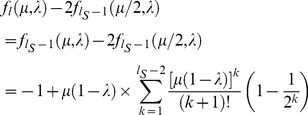
(25)and so this expression is equal to 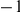 for 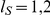, and then increases with successive values of 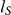.

Now, because 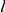 is re-set to 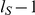 for 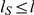, it follows that we take 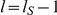 for 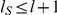. For 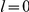, we then obtain that 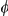 is maximized over 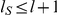 for 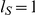, while when 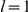, we obtain that 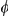 is maximized over 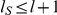 for 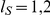. For 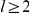, we obtain that 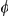 is maximized over 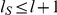 for 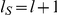.

Therefore, in any case, we can maximize 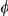 over 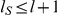 by taking 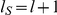. Since we can maximize 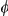 over 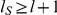 by setting 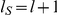, it follows that 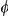 is maximized when 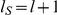.

We reach the conclusion that, when the fitness penalty for having a non-viable genome is sufficiently great, *the SOS response will confer a maximum selective advantage if it is activated when and only when the genome has sustained sufficient genetic damage so that it will be unviable without SOS repair*. However, it should be emphasized that this only holds when 
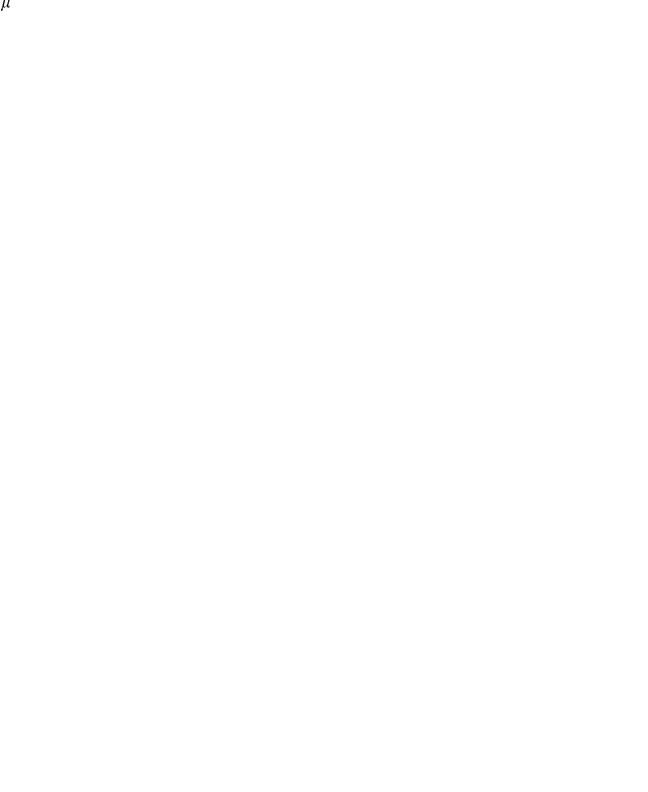
 is not near the error catastrophe, so that 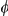 is sufficiently larger than 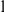 for large 
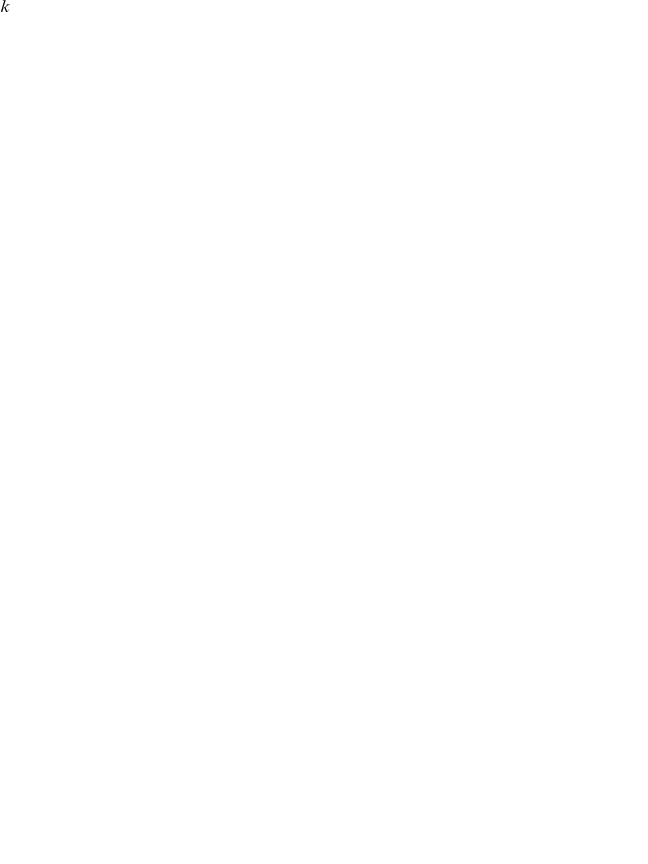
 that the above analysis holds.

### Stochastic Simulations

We developed stochastic simulations of a unicellular population capable of undergoing the SOS response, in order to numerically test the analytical predictions of our model. We consider a constant population of genomes that is cycled over every time step. During each cycle, every genome is allowed to replicate with a probability 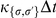, where 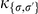 is the first-order growth rate constant of genome 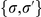, and 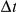 is the length of the time step. We take 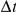 to be sufficiently small so that the probability of a given genome replicating more than once during a cycle is negligible.

We assume that the population initially consists of a clonal population of wild-type (mutation-free) genomes. The fitness of a given genome 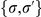 is determined by assigning 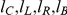 parameters to the ordered-pairs 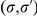, 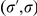 with respect to the ordered-pair 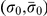. For each set of 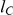, 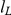, 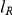, and 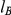 parameters, a fitness is assigned based on the fitness landscape defined previously. The fitness of the genome is then taken to be the larger of the two calculated fitnesses. In the limit of infinite sequence length, this prescription for calculating fitnesses becomes identical to the method used in the analytical solution of our model.

If a genome replicates during a cycle, then it is removed from the population, and the two daughters are added to the population of genomes. To maintain a constant population size, another, randomly chosen genome is removed from the population as well. Because this approach is simply the stochastic implementation of the quasispecies dynamics of the system, it converges to the infinite population, continuous time result as the population size gets larger and the time steps get smaller.

If a daughter genome is produced that has at least 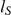 lesions, then it enters the SOS response, and is assigned a replication probability of 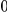. A genome that has initiated the SOS response continues to undergo SOS repair until all lesions have been removed, and a complementary genome has been restored. During every time step, a genome that is undergoing the SOS response has its lesions scanned, and each lesion is repaired with probability 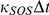. In addition to being chosen small enough so that the probability of a given genome replicating more than once during a cycle is negligible, we also choose 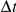 to be sufficiently small so that the probability that a given genome undergoing the SOS response has more than one lesion repaired during a cycle is also negligible.

The stochastic simulation is allowed to run for a sufficient number of time steps so that the mean fitness of the population does not change significantly, at which point the system is assumed to be at steady-state.

**Figure 2:**
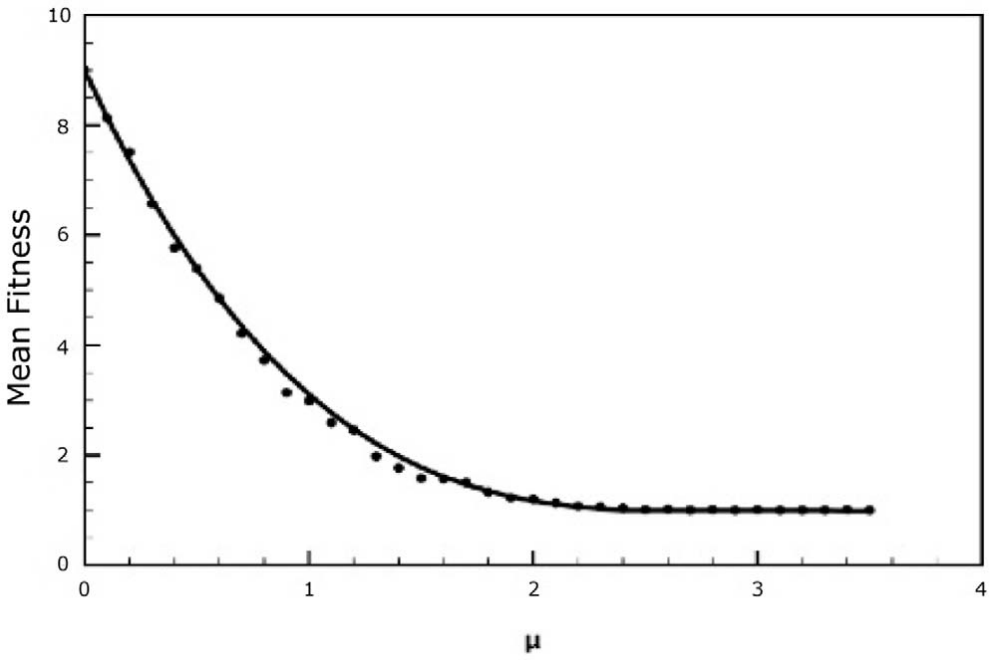
Comparison of the mean fitnesses obtained from both stochastic simulations (dots) and the analytical solution (solid line) of our model. Parameter values are 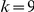, 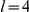, 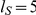, 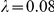, 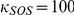, 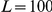. The population size was set at 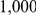.

**Figure 3:**
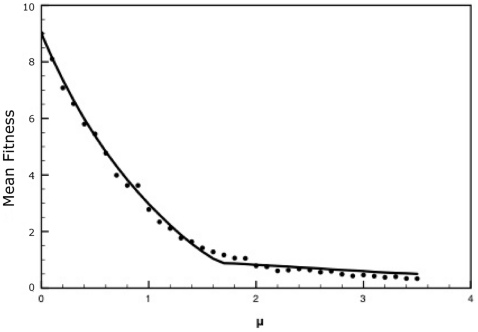
Comparison of the mean fitnesses obtained from both stochastic simulations (dots) and the analytical solution (solid line) of our model. Parameter values are 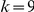, 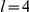, 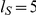, 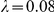, 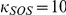, 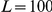. The population size was set at 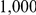.

Figures 2 and 3 show plots comparing the mean fitness obtained from the analytical solution to the mean fitness obtained from the stochastic simulations. As can be seen from the figures, the agreement between the analytical solution and the stochastic simulation is excellent.

### Conclusions and Future Research

This paper developed a quasispecies approach for describing the evolutionary dynamics of a unicellular population that incorporated a simplified model of the SOS response. The model was a generalization of the single-fitness-peak landscape that is often used in quasispecies theory to study various problems in evolutionary dynamics. The model was shown to be analytically solvable, and it was found that the solution led to a maximal selective advantage to the SOS response in a manner that is broadly consistent with the behavior of actual organisms. Specifically, we showed that the SOS response should only be activated in a cell with a sufficiently damaged genome that it will be unviable if the SOS response is not activated. In such a situation, the cell has “nothing to lose,” meaning that it is better to attempt to repair the genome and risk introducing deleterious mutations, than it is to leave a highly damaged genome alone.

For future research, it will be important to consider more realistic models that will allow for quantitative models that can be used in collaboration with experiment. Because the SOS response is a genetic repair pathway that works in conjuction with other cellular repair pathways, a proper understanding of the SOS response is important for developing a coherent theory of mutation-propagation that will be useful for understanding the emergence of antibiotic drug resistance in bacteria, and cancer in multicellular organisms [2]–[4].
